# Obituary

**Published:** 1880-09

**Authors:** 


### OBITUARY.

Died—In Vineville, Ga., on Saturday, July 28, of consumption, Dr. James T. Holmes, recently of Albany, Ga.

Dr. Holmes was one of the prominently rising members of the profession of the South. Though comparatively young, he had already made a reputation of which an older man might well be proud. He was interested in every work that would promote the interests of his chosen profession. He was a graduate of the Ohio College of Dental Surgery, and has ever been an ardent friend of the institution. He was beloved by all who knew him, and in his demise the profession and society have lost a valuable member. The family will have the sincere sympathy of all who knew him.

